# Type 2 Diabetic Mellitus Inhibits Skin Renewal through Inhibiting WNT-Dependent Lgr5+ Hair Follicle Stem Cell Activation in C57BL/6 Mice

**DOI:** 10.1155/2022/8938276

**Published:** 2022-04-16

**Authors:** Minghui Wang, Shangsheng Yao, Dehua He, Mulan Qahar, Jinqing He, Meifang Yin, Jun Wu, Guang Yang

**Affiliations:** ^1^Department of Burn and Plastic Surgery, Shenzhen Institute of Translational Medicine, Shenzhen Second People's Hospital, The First Affiliated Hospital of Shenzhen University Health Science Center, Shenzhen 518035, China; ^2^Human Histology & Embryology Section, Department of Surgery, Dentistry, Pediatrics & Gynecology, University of Verona Medical School, Verona 37134, Italy

## Abstract

**Background:**

Hair follicles are important accessory organs of the skin, and it is important for skin renewal and performs variety of important functions. Diabetes can cause several dermatoses; however, its effect on hair follicles is unclear. The purpose of this study was to investigate the effect of type II diabetes (T2DM) on the hair follicles of mice.

**Methods:**

Seven-week-old male C57BL/6 littermate mice were divided into two groups. The treatment group was injected with streptozotocin (STZ) to induce T2DM, and the control group was parallelly injected with the same dose of buffer. Seven days after injection, the back is depilated to observe the hair follicle regeneration. Hair follicle regeneration was observed by naked eyes and HE staining. The proliferation of the skin cells was observed by PCNA and K14 staining. The altered genes were screened by RNA sequencing and verified by qRT-PCR. In addition, Lgr5 + GFP/mTmG transgenic mice were used to observe the effect of T2DM on Lgr5 hair follicle stem cells (HFSC). And the expression of WNT4 and WNT8A were measured by Western Blot.

**Results:**

T2DM inhibited hair follicle regeneration. Compared to control mice, T2DM mice had smaller hair follicles, reduced skin thickness, and less expression of PCNA and K14. RNA sequencing showed that the two groups had significant differences in cell cycle and proliferation-related pathways. Compared with the control mice, the mRNA expression of Lgr4, Lgr5, Wnt4, and Wnt8a was decreased in the T2DM group. Moreover, T2DM inhibited the activation of Lgr5 HFSC and the expression of WNT4 and WNT8A.

**Conclusions:**

T2DM inhibited hair follicle regeneration and skin cells proliferation by inhibiting WNT-dependent Lgr5 HFSC activation. This may be an important reason for the reduction of skin renewal ability and the formation of chronic wounds caused by diabetes. It is important for the treatment of chronic diabetic wounds and the development of tissue engineering.

## 1. Introduction

Diabetes affects more than 340 million people worldwide, and about one-third of patients are accompanied by skin disorders [1, 2]. Hyperglycemia causes damage to a wide range of skin cell populations, including endothelial cells, keratinocytes, fibroblasts, and neurons. Diabetic ulcers are one of the common complications of diabetes, and about 20% of patients are affected by diabetic ulcers [3]. Diabetic ulcer is difficult to heal and is usually accompanied by infection, which causes a significant economic burden to the patients [4]. The most common diabetic ulcer is diabetic foot, and about 6% of diabetic patients have different degrees of foot infection and ulcers [5]. Between 0.03% and 1.5% of patients require amputation because of long-term ulcers [6]. At present, the most useful methods to prevent foot complications are foot care and screening [7]. Until now, there is no effective medication or method for the treatment of diabetic ulcers.

The skin is the first line of defense against external aggressions. The keratinocytes on the skin surface form a natural barrier, which can prevent the invasion of various microorganisms and physical and chemical substances. There are various stem cell populations in the skin, and they maintain the skin's stability to resist the external environment by continuously proliferating new cells to replace the aging skin cells [8]. When the cell proliferation capacity decreases, the skin renewal rate will decrease. Undoubtedly, when the cell proliferation rate cannot follow the skin renewal requirements, it will increase the ulcers probability and decrease wound healing. Previous studies have confirmed that diabetes can cause a variety of skin complications [1, 9]. In vivo studies have shown that hyperglycemia can cause epidermal dysfunction, accelerate skin aging, inhibit the proliferation and differentiation of keratinocytes, and increase skin cell apoptosis [10–12]. In vitro studies also proved that high-glucose environment can inhibit the differentiation and function of human immortal keratinocyte line (HaCaT), inhibit the viability of fibroblasts, and reduce the migration of keratinocytes [1, 13–15]. These studies have provided sufficient evidence for diabetes to reduce skin renewal capacity. The classical opinion believes that diabetic ulcers are usually caused by neuropathy, insufficient blood supply, and infection [3]. However, the concept that the formation of diabetic ulcers is associated with reduced skin renewal capacity has not been widely accepted. Stronger evidence is needed to support this view.

Hair follicle is a complex micro-organ in the dermis. When the skin is injured, the epidermal stem cells in the hair follicle are activated and migrated to the wound site and then differentiated into epidermal cells, which contributes to the reepithelialization of the wound [16, 17]. Mice with defective hair follicle development showed a significant delay in reepithelialization [18, 19]. Hair follicles are one of the deepest components in the skin. The activation of hair follicle stem cells (HFSCs) helps to repair non-full-thickness skin injury, allowing missing skin to regenerate from hair follicles. Leucine-rich repeat-containing G protein-coupled receptor (Lgr5) is one of the biomarkers of HFSCs [20, 21]. The activation of Lgr5/Wnt/*β*-catenin signaling pathway is the key to hair follicle regeneration. Lgr5 depletion inhibits hair follicle regeneration [22]. This phenomenon is reversible due to the transdifferentiation between stem cells [22]. In addition, the activation of HFSCs determines the hair follicle cycle. The hair follicle cycle includes anagen, catagen, and telogen [23, 24]. The anagen (active growth phase) is the period when the hair follicles grow most vigorously. At the same time, activated melanocytes secrete melanin to defend against light radiation. During the catagen (transition phase), follicle matrix cells stop proliferating and melanocytes stop secreting melanin. The size of the hair follicle begins to decrease. During the telogen (resting phase), the hair follicle shrinks further, and the hair begins to fall out. When the body needs it, the hair follicles retransition from the telogen to the anagen. Based on our experience, the hair follicles of C57BL/6 mice transit from the anagen to the telogen when they are 6-8 weeks old. Hair regrowth could be observed 10-14 days after the back is depilated, and the skin turns from white to bluish-black, which means that the HFSCs start to proliferate. After 21 days, the new hair will be the same length as the original hair.

Recently, hair follicle transplantation is considered a promising method for the treatment of diabetic ulcer. Traditional hair follicle transplantation collects hair follicles from the occipital region and transplants them to the bald area. The transplanted hair follicles will regenerate new hair within a few months [25]. Recent clinical studies show that autologous hair follicle transplantation can promote wound healing, especially diabetic ulcers [26–28]. For example, hair follicle transplantation can promote the diabetic leg ulcers healing and vascular regeneration [29]. Transplantation of epidermal sheets derived from hair follicles can accelerate chronic wound healing in patients with diabetes and chronic venous insufficiency of the lower extremities [30, 31]. Compared with abdominal skin without hair follicles, head skin with hair follicles accelerates the diabetic ulcer healing [32]. In addition, hair follicle transplantation can promote the wound healing of autosomal recessive dystrophic epidermolysis bullosa [33]. These studies not only prove the importance of hair follicles in maintaining skin regeneration, but also prove that hair follicles have great application potential as a tissue engineering biomaterial.

Diabetes affects skin regeneration, but the effect of diabetes on hair follicles is controversial. Only a few studies have put forward the idea that diabetes is associated with hair loss, but there is a lack of experimental proof [34–36]. We hypothesize that diabetes reduces the skin renewal capacity by inhibiting the hair follicles regeneration. Therefore, to observe the effect of diabetes on the hair follicles, we used streptozotocin (STZ) to induce type II diabetes (T2DM) in mice and observed the hair follicles regeneration on the back of the mice and explored the underlying mechanisms.

## 2. Materials and Methods

### 2.1. Ethical

The study related to animal use has been complied with all the relevant national regulations and institutional policies for the care and use of animals and has been approved by the Animal Care and Use Committee of Shenzhen Second People's Hospital.

### 2.2. Animals

Male C57BL/6 mice were purchased from Charles River (Beijing, China). Lgr5 + GFP/mTmG mice, which show green fluorescence in LGR5 protein and red fluorescence in the cell membrane, were gifts from Prof. Wang Xusheng of Sun Yat-sen University (Guangzhou, China). Animals were housed at the animal center of the Shenzhen Institute of Translational Medicine, under constant temperature (22-26°C) and half-day light/dark cycle schedule with free access to food (1025; HFK, Beijing, China) and water. Mice were anaesthetized using isoflurane (970-00026-00; RWD, Shenzhen, China).

After fasting for 16 hours, 20 mice were injected with STZ (120 mg/kg, dissolved in 0.028 mol/L citric acid and 0.022 mol/L sodium citrate buffer with pH 4.4) to induce T2DM [37]. Blood glucose levels were checked during the 2nd and 7th day after the injection. Mice with blood glucose ≥16.7 mmol/L were included in the experiment. On the 7th day of the experiment, hair on the back was removed with Veet® hair removal cream and then continued to observe the hair regrowth and hair follicle cycle transition.

### 2.3. Histology

At execution, mice were perfused with PBS. Skin samples were fixed in 4% paraformaldehyde (PFA) overnight and sent to Servicebio (Wuhan, China) for paraffin fixation services. Five *μ*m slides were stained using a hematoxylin-eosin (H&E) kit (Servicebio, Wuhan, China). Pictures were taken under a microscope scanning system (SQS-40P, Shengqiang, Shenzhen, China).

### 2.4. Immunohistochemistry

For PCNA staining, the slides were incubated at 65°C for 2 h and dewaxed with xylene (15 min, 15 min), alcohols (100% 5 min, 100% 3 min, 95% 3 min, 80% 3 min), and water (5 min, 5 min). Slides were soaked in a pH 6.0 citrate solution at 95°C for 12 min and naturally cooled for 30 minutes. Next, the slides were incubated with endogenous peroxidase blocker (Kit-7310 reagent 1, Maixin, Fuzhou, China) for 15 min at 37°C, nonspecific staining blocker (Kit-7310 reagent 2, Maixin, Fuzhou, China) for 60 min, anti-PCNA antibody (A0264, ABclonal, Wuhan, China) overnight at 4°C, biotin-labeled goat anti-mouse/rabbit IgG polymer (Kit-7310 reagent 3, Maixin, Fuzhou, China) for 30 min at 37°C, and streptavidin-peroxidase (Kit-7310 reagent 4) for 15 min at 37°C. Diaminobenzidine (DAB, DAB-1031, Maixin, Fuzhou, China) staining is used for the brown color and hematoxylin staining for the background. Seal the slides with neutral gum.

### 2.5. Immunofluorescence

To assess cell proliferation, we used the immunohistochemistry kit (Kit-7310, Maixin, Fuzhou, China). Briefly, slides were incubated with 3% H_2_O_2_ for 30 min at 37°C, nonspecific staining blocker for 60 min, anti-K14 antibody (10143-1-AP, Proteintech, Wuhan, China) overnight at 4°C, HRP secondary antibody (ab150165, Abcam, Cambridge, UK) for 60 min 37°C, and DAPI (G1235-4, Servicebio, Wuhan, China). Pictures were taken under a microscope (Revolve FL, Discover echo, San Diego CA, USA).

### 2.6. RNA Sequencing

Samples were preserved in RNALater® at 4°C overnight. Then, samples were stored in dry ice and sent to the RNA-sequencing company (Nuomi, Suzhou, China). The data were processed by cluster heatmap, volcano map, Gene Ontology (GO), Reactome, and Kyoto Encyclopedia of Genes and Genomes (KEGG) enrichment analysis through the company technicians.

### 2.7. qRT-PCR

RNA samples were returned from the company after sequencing and transcribed to complementary DNA (cDNA) by synthesis kit (K1622, Thermo, Waltham MA, USA). Quantitative real-time PCR (qRT-PCR) kit was purchased from Bimake (B21203, Bimake, Shanghai, China). All the steps were performed according to the manufacturer's instructions. The qRT-PCR analysis was performed on an ABI-Q3 apparatus (ABI, Foster, USA) with normalization to *Gapdh* as the reference gene. Details were described as before [38, 39]. Primers sequences: *Lgr4*, forward 5′-CCCGACTTCGCATTCACCAA-3′, reverse 5′-CCTGAGGAAATTCATCCAAGTT-3′; *Lgr5*, forward 5′- CCTACTCGAAGACTTACCCAGT-3′, reverse 5′-GCATTGGGGTGAATGATAGCA-3′; *Wnt4*, forward 5′- AGACGTGCGAGAAACTCAAAG-3′, reverse 5′- GGAACTGGTATTGGCACTCCT-3′; *Wng8a*, forward 5′-GGGAACGGTGGAATTGTCCTG -3′, reverse 5′-GCAGAGCGGATGGCATGAA -3′; *Gapdh*, forward 5′-AGGTCGGTGTGAACGGATTTG -3′, reverse 5′-GGGGTCGTTGATGGCAACA -3′.

### 2.8. Western Blot

Details have been previously described [40]. Proteins were detected by anti-WNT4 and anti-WNT8A (GB112192, GB112250, Servicebio, Wuhan, China) rabbit polyclonal antibodies. Anti-*β*-TUBULIN mouse monoclonal antibody was obtained from Servicebio (GB13017-2).

### 2.9. Statistics

Data are reported as mean ± standard deviation (SD). Group size assayed in each experiment is indicated in the figure legends. Group mean differences were analyzed by Student's *t*-test using GraphPad Prism 6 (San Diego, USA). A *P* < 0.05 was considered statistically significant.

## 3. Results

### 3.1. T2DM Inhibits Hair Regrowth and Reduces Skin Thickness

**Figure 1:**
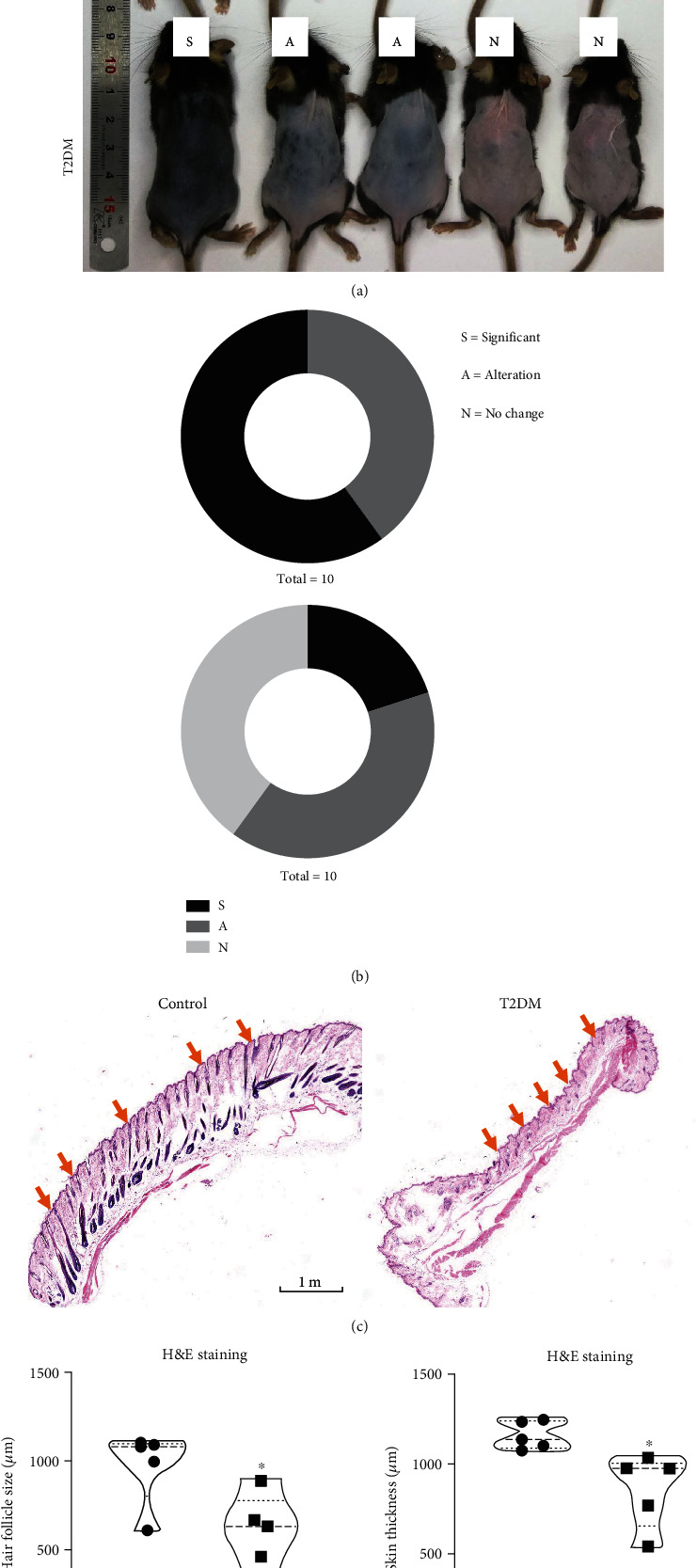
T2DM inhibits hair cycling and reduces skin thickness. (a) Representative photo of hair regrowth on the back of mice. S: significant; A: alteration; N: no change. (b) 40% and 60% of control mice show significant hair regrowth and hair follicle alteration, respectively. 20% and 40% of T2DM mice show significant hair regrowth and hair follicle alteration, respectively. The other 40% of mice have no change. (c) Representative photos of hair follicles. Orange arrows direct the hair follicles. (d) T2DM significantly decreased hair follicle size (control 975 ± 210 vs. T2DM 568.8 ± 254.3*μ*m, *P* = 0.0249, *n* = 5) and skin thickness (control 1159 ± 77.69 vs. T2DM 859.8 ± 204.4*μ*m, *P* = 0.0155, *n* = 5). ∗*P* < 0.05.

Mice were depilated on day 7 after successful induction of T2DM. As expected, on the 10th day after depilation, there was a significant difference in hair regrowth between the two groups. Compared to control group, T2DM inhibited hair regrowth (Figures 1(a) and 1(b)). To further confirm the inhibitory effect of T2DM on hair follicles, we observed skin tissue sections. The H&E staining showed that T2DM not only inhibited the hair follicles regeneration, but also reduced the skin thickness (Figures 1(c) and 1(d)). It means that T2DM may delay the hair follicles cycle transition and inhibit skin cells proliferation. Apart from this, we did not observe any changes in other skin structures and organs.

### 3.2. T2DM Inhibits the PCNA Expression and K14 Proliferation

**Figure 2:**
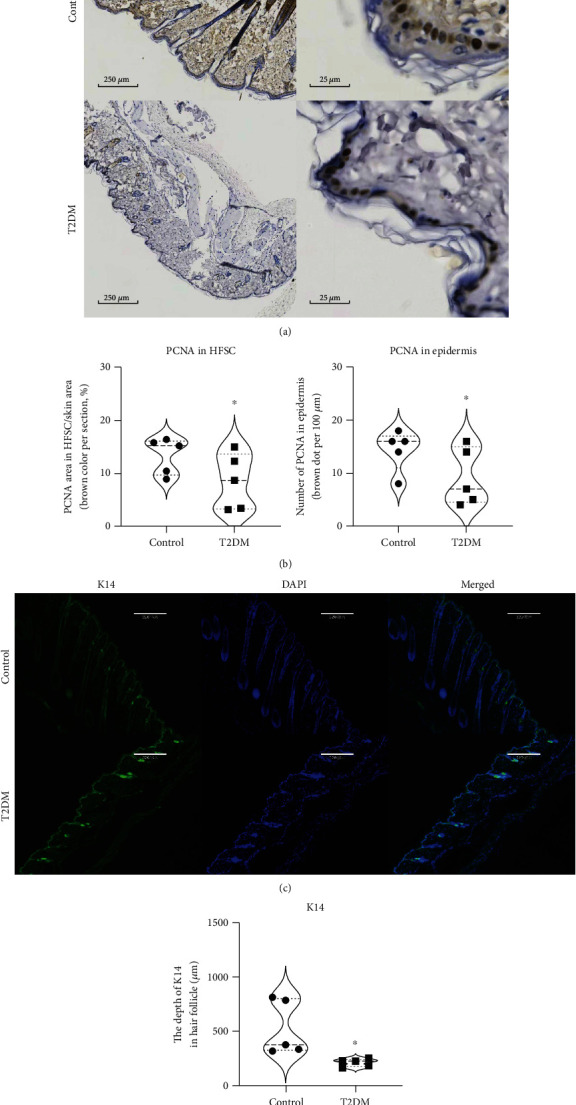
T2DM inhibits skin cells proliferation. (a) Representative photos of PCNA in the skin. (b) T2DM decreased the PCNA expression in hair follicles (control 13.31 ± 3.428 vs. T2DM 8.462 ± 5.272, brown area %, *P* = 0.0204, *n* = 5) and epidermis (control 14.4 ± 3.387 vs. T2DM 9.2 ± 5.45, brown dot per 100 *μ*m, *P* = 0.0387, *n* = 5). (c) Representative photos of K14 and DAPI staining in skin. (d) T2DM reduced the depth of K14 in hair follicles (control 525.8 ± 250.5 vs. T2DM 212.2 ± 36.23, *μ*m, *P* = 0.0353, *n* = 5). ∗*P* < 0.05.

To study whether T2DM inhibited the hair follicles regeneration and skin cells proliferation, we observed the expression of PCNA and K14, which are important biomarkers of cell proliferation. T2DM inhibited the expression of PCNA in hair follicles and epidermis (Figures 2(a) and 2(b)). Also, T2DM reduced the fluorescence intensity of K14 (Figures 2(c) and 2(d)). This shows that T2DM can inhibit hair follicles regeneration and skin cells proliferation.

### 3.3. T2DM Inhibits HFSCs Activation and Cell Cycle-Related Pathways

**Figure 3:**
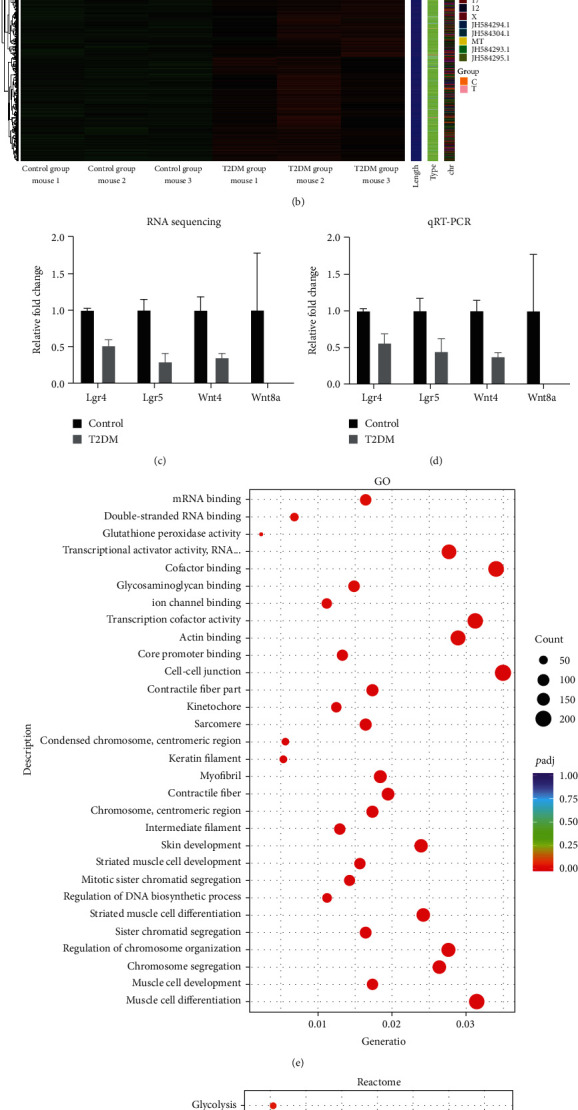
RNA sequencing results. (a) Volcano map. (b) The mRNA expression levels between samples. (c, d) Lgr4, Lgr5, Wnt4, and Wnt8a expression levels in RNA sequencing and qRT-PCR, respectively. (e, f, g) GO, Reactome, and KEGG bioinformatic analysis results.

To explore the underlying mechanism of T2DM inhibited skin cells proliferation and hair follicles regeneration, we analyzed the differences in mRNA expression between the two groups. The results showed that there were significant differences in mRNA expression between the two groups (Figures 3(a) and 3(b)). Moreover, the bioinformatics analysis of GO, Reactome, and KEGG showed significant differences in the expression of genes related to cell cycle, proliferation, and division (Figures 3(e), 3(f), and 3(g)). Compared with the control group, T2DM significantly inhibited the expression of *Lgr4*, *Lgr5*, *Wnt4*, and *Wnt8a* genes, which are related to the activation of HFSCs (Figure 3(c)). The results of qRT-PCR are consistent with the results of RNA sequencing (Figure 3(d)). These results suggest that T2DM inhibits the proliferation of skin cell populations by inhibiting the cell cycle. And it is closely related to LGR5/WNT pathway-dependent HFSCs activation.

### 3.4. T2DM Inhibits Lgr5+ Hair Follicle Stem Cells Activation and WNT Expression

**Figure 4:**
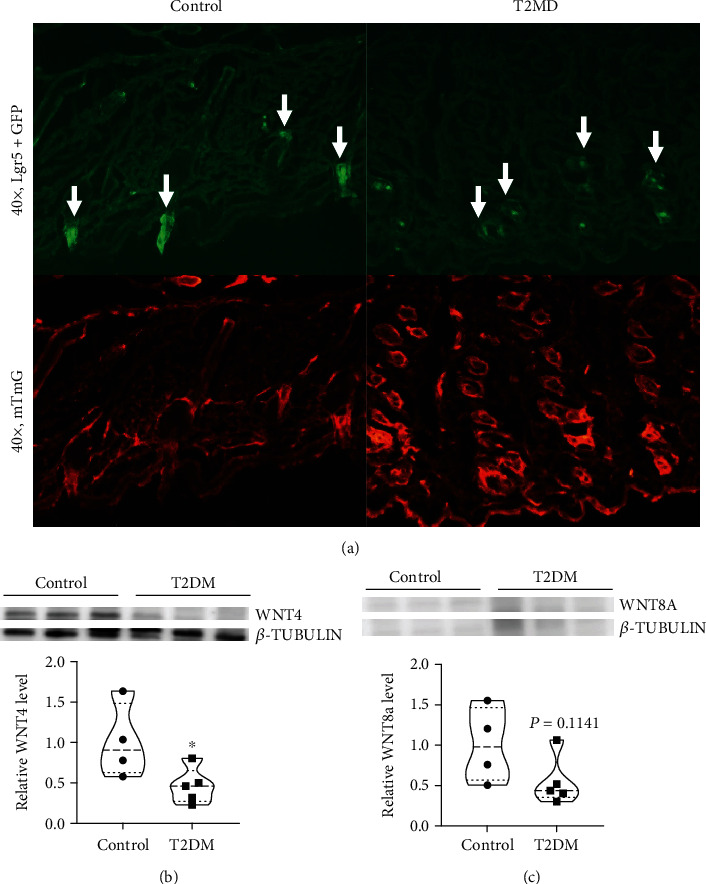
T2DM inhibits Lgr5+ hair follicle stem cells activation and WNT expression. (a) Representative photos of skin sections from Lgr5 + GFP/mTmG mice. (b, c) WNT4 (control 1 ± 0.4579 vs. T2DM 0.4562 ± 0.2201, *P* = 0.05, *n* = 4 − 5) and WNT8A (control 1 ± 0.4648 vs. T2DM 0.5404 ± 0.3002, *P* = 0.1141, *n* = 4 − 5) protein levels in skin. ∗*P* < 0.05.

To identify whether T2DM inhibited WNT-dependent Lgr5 hair follicle stem cell activation, we used Lgr5 + GFP/mTmG mice. Compared with the control mice, the green fluorescence of the T2DM mice was significantly reduced as shown in Figure 4(a). Similarly, T2DM suppressed the expression of WNT4 protein (Figure 4(b)). However, the level of WNT8A protein was not significantly different between the two groups, which may be related to the lower group size and lower protein expression level (Figure 4(c)).

## 4. Discussion

The cause of diabetic ulcers has been attributed to neuropathy, insufficient blood supply, and infection [3]. Here, it is the first time shown that T2DM inhibits the skin renewal capacity by inhibiting the WNT-dependent Lgr5 hair follicle stem cells activation. In addition, T2DM directly inhibits cutaneous cells proliferation, including K14 and HFSCs, by inhibiting the cell cycling. This may be a new mechanism for the formation of diabetic ulcers. This study not only explains why T2DM affects skin regeneration, but also gives a new way for the development of treatments for diabetic ulcers in the future.

There is no doubt that high glucose affects cells activity and physiological functions. For example, high glucose can inhibit the differentiation of neural stem cells [41] and also lead to the death of cardiac stem cells [42]. In this study, T2DM inhibited the activation of Lgr5 HFSC, the proliferation of K14, and the production of PCNA, which means that T2DM reduced the renewal capacity of the skin. The following RNA sequencing results further proved our hypothesis that the T2DM inhibited cell cycling. Because HFSCs can differentiate into a variety of skin cells, we believe that T2DM inhibits the skin renewal capacity by inhibiting the Lgr5 HFSCs activation.

Hair follicles are distributed in most areas of the body, but there are differences in distribution and morphology [43]. In humans, most of the hair on the body surface is small and colorless, while the hair on the head is longer and denser. The hair follicles in the same area may also have significant characteristics differences in different districts, such as the top of the head and the headrest area. Compared with the hair follicles on the top of the head, the hair follicles of the headrest have stronger environmental adaptability and can resist androgenetic alopecia [44]. Therefore, the hair follicles in the headrest area are also considered as premium donor sites for hair follicle transplantation [45]. Similarly, it is considered that the skin from the head is more suitable to be a donor site for skin grafting. It can be explained that the headrest contains high-quality hair follicles, which increases the success rate of skin grafting. In contrast, hair follicles from nonpremium donor sites may have reduced proliferation and differentiation capacity due to environmental interference. As far as we know, Balb/c background nude mice have no hair in most areas of their bodies, but a small amount of hair growth can be observed on the head and beard. This study focused the effect of T2DM on hair follicles from the back of mice. As expected, these hair follicles are sensitive to diabetes. This can explain why diabetic patients have reduced skin renewal capacity and are more likely to form chronic wounds. Nevertheless, diabetic ulcers rarely occur on the head, and hair loss is not yet considered a symptom of diabetes. Unfortunately, the effect of diabetes on hair follicles of the head and the limbs in mice has not been studied yet. This is also an important area to research upon in the future.

In addition, the demand for glucose of hair follicles from different regions is inconsistent. Generally, the growth of hair follicles on the head has a higher energy requirement, which is related to the metabolism of glucose and glucose derivatives [46, 47]. Moreover, the glucose sensitivity of stem cells activation and differentiation are inconsistent [48, 49]. For example, muscle stem cells have different requirements for glucose during proliferation, resting, and differentiation [50]. However, we have not found any research on the relationship between hyperglycemia and trunk hair follicles. At least, our research supports the result that trunk hair follicles are sensitive to hyperglycemia.

Skin grafting is the golden standard for the treatment of large-area burns and full-thickness wounds [51–53]. The success rate is related to problems, such as poor grafts, infections, and donor site morbidity [52, 54]. For diabetic patients, skin grafting may also cause new wounds, which may have complications and difficult to heal. For patients with skin defects and burns that exceed 50%-60% of the total body surface, autologous skin grafting is impractical due to insufficient donor sites [53, 55]. Although autologous skin grafting is the first choice, in some cases, artificial skin is also a good choice. Besides, hair follicle transplantation can provide a source of autologous keratinocytes for wound healing. Hair follicles can be obtained quickly and will not cause large wounds. Therefore, hair follicle transplantation has great potential in the treatment of diabetic ulcers. In addition to this, hair follicles can be proliferated through the hair follicle recombination technology in vitro, and it can be used in the treatment of patients with severe burns or diabetes. It does not only reduce the need for donation area, but also reduces the risk of infection. Recently, bioprinting technology has provided a good choice for wound repair. It does not only print 3-dimensional bionic skin, but also create a stable structure. Although there are more and more biomaterials that combine stem cells with bioprinting, there is no biomaterial containing hair follicles and HFSCs [53]. The development of biomaterials containing HFSCs or hair follicle structure has potential application prospects and clinical value.

We were interested to publish this study as soon as possible; therefore, this study has some limitations. First, the group size is small, and we will increase the group size to confirm the accuracy and universality of the research. Second, we used male mice, and we are planning to use female mice as well in our future study. Third, hair follicles in mice are different from those in humans. It is not sure whether the phenomenon that diabetes inhibits hair follicle regeneration also exists in humans. And it is uncertain alopecia is related to diabetes; therefore, further research is needed. Fourth, our research period is short. We only observed hair follicle and skin changes within 3 weeks after T2DM induction. We did not observe changes in other skin structures during this study period. Diabetes is known to cause chronic organ injury. Longer study periods are required if the long-term effects of T2DM on the skin are to be observed. Lastly, our research only explains the phenomenon and mechanism of T2DM inhibiting the regeneration of hair follicles on the back of mice, and the rest of the site is still unclear. We are planning to add more data in the future.

## 5. Conclusions

In summary, T2DM inhibits skin self-renewal by inhibiting WNT-dependent Lgr5 HFSCs activation. It may be a key factor in the formation of diabetic chronic wounds. This study not only provides a new explanation for the formation of diabetic chronic wounds, but also provides a new theoretical basis for hair follicle transplantation. Finding a mechanism that can resist the HFSCs inhibitory effects of diabetes may lead to the development of new drugs.

## Data Availability

The data used to support the findings of this study are available from the corresponding author upon request.
